# EPS15-AS1 Inhibits AKR1B1 Expression to Enhance Ferroptosis in Hepatocellular Carcinoma Cells

**DOI:** 10.7150/jca.89993

**Published:** 2024-01-01

**Authors:** Quan Man, Guoyou Zhang, Xiaojun Chen, Sa Ren Na, Siguleng Bai, Haoqiang Zhi, Ling Sun, Huifang Pang

**Affiliations:** 1 General Surgery, Tongliao City Hospital, Tongliao, Inner Mongolia, 028000, China.; 2 Department of Gastroenterology, Tongliao City Hospital, Tongliao, Inner Mongolia, 028000, China.; 3 Department of Pathology, Tongliao City Hospital, Tongliao, Inner Mongolia, 028000, China.

**Keywords:** EPS15-AS1, AKR1B1, hepatocellular carcinoma, ferroptosis

## Abstract

Epidermal growth factor receptor substrate 15 (EPS15) is part of the EGFR pathway and has been implicated in various tumorigenesis. Increasing evidence suggests that long noncoding RNA (lncRNA) plays an essential role in liver hepatocellular carcinoma (LIHC) by regulating the expression of proteins and genes. Through analysis of the cancer genome atlas (TCGA) database, we found that EPS15 is highly expressed in LIHC tissue, and lncRNA EPS15-antisense1 (EPS15-AS1) decreased in LIHC cell lines. However, the function of EPS15-AS1 in LIHC is still unknown. When EPS15-AS1 was overexpressed in HepG2 cell lines, the expression of EPS15 was reduced and cell activity and invasiveness were inhibited. In addition, we observed an increase in Fe^2+^ ion and lipid peroxidation after overexpression of EPS15-AS1, and further analysis showed that the susceptibility to ferroptosis increased. Aldo-keto reductase family 1 member B 1 (AKR1B1) belongs to the aldo/keto reductase superfamily and is involved in maintaining the cellular redox balance. Survival analysis revealed that patients with a higher level of AKR1B1 have a lower survival rate in the TCGA database. We also found that EPS15 enhanced the AKR1B1 expression in LIHC, and AKR1B1 had the ability to promote cell invasiveness. Moreover, overexpression of AKR1B1 alleviated the promoting effect of EPS15-AS1 on ferroptosis. Therefore, EPS15-AS1 can induce ferroptosis in hepatocellular carcinoma cells by inhibiting the expression of EPS15 and AKR1B1 and disrupting the redox balance. EPS15 and AKR1B1 may serve as biomarkers for diagnosis and lncRNA EPS15-AS1 potential drug for LIHC.

## Introduction

Liver hepatocellular carcinoma (LIHC) is the most common type of primary liver cancer, accounting for 90% of liver cancers 1, 2. Chronic infection due to hepatitis B and C viruses is a common risk factor for LIHC, which has become the cancer with the highest recurrence rate worldwide 1-3. Additionally, obesity, diabetes, alcohol consumption, and other risk factors for liver injury can further promote the development of LIHC 3, 4. The etiology of LIHC is closely related to environmental factors and requires adaptation to changing environmental conditions, in which epigenetic aberrations play a critical role in the development and progression of LIHC 4. DNA methylation and acetylation, alterations in microRNAs and long noncoding RNAs (lncRNAs), and chromatin modifications are the most common epigenetic modifications that also lead to changes in the liver epigenome 4, 5. The accumulation of these epigenetic alterations leads to carcinogenesis, progression, and metastasis. LncRNAs are defined as noncoding RNAs greater than 200 nucleotides in length 6. LncRNAs mainly include enhancer RNAs, sense or antisense transcripts, and intergenic transcripts 6, 7. LncRNAs are thought to have multiple functions, including the organization of nuclear structural domains, transcriptional regulation, and regulation of protein or RNA molecules 7. However, the biological processes of the vast majority of lncRNAs remain unknown.

Receptor tyrosine kinases (RTKs) are a family of signaling proteins in which growth factor RTK-mediated cell signaling pathways are essential in maintaining normal physiological functions 8. However, their aberrant activation promotes tumor development 9. Currently, epidermal growth factor receptor (EGFR) is one of the most studied RTK signaling proteins and is closely associated with the development of multiple human tumors 10, 11. The epidermal growth factor receptor pathway substrate 15 (EPS15) was originally identified as a substrate for the EGFR signaling pathway 12. Notably, in acute myelogenous leukemias, the EPS15 gene was found to rearrange at t (1;11) (p32, q23), suggesting a role for EPS15 in tumorigenesis and development 13. In addition, Eps15 was also found to be involved in endocytosis and cell growth regulation 14. Therefore, EPS15 may affect the signaling efficiency of EGFR and be involved in the development of some tumors.

LncRNAs can be categorized into five classes, based on their relative position to nearby coding genes: antisense lncRNAs, intronic lncRNAs, intergenic lncRNAs, bidirectional lncRNAs, and promoter-associated lncRNAs, which regulate genes expression in very different ways 7, 15. Antisense lncRNAs are transcribed from the antisense strand of a gene (usually a protein-coding gene) and overlap with the mRNA of the gene 15. The presence and positional specificity of this naturally occurring antisense lncRNA suggest that it tends to act more closely with the sense strand than with target genes in general 16. According to the current study, the mechanisms by which AS-lncRNAs affect gene expression on the sense strand can be divided into three categories 16: 1) The transcription process of AS-lncRNAs represses sense-strand gene expression. 2) AS-lncRNAs bind to DNA or histone-modifying enzymes and regulate the epigenetics of sense-strand genes, thereby affecting gene expression. 3) AS-lncRNAs bind to sense-strand mRNA through base complementary pairing and affect variable splicing of mRNA, thereby affecting protein translation and function. LncRNA EPS15-antisense1 (EPS15-AS1) is an antisense lncRNA of EPS15, which has been reported to inhibit EPS15 expression and induce apoptosis 17. However, the role of EPS15-AS1 in LIHC and the mechanism are still unclear.

Ferroptosis is a novel type of programmed cell death triggered by iron-dependent lipid peroxidation, ultimately leading to cell membrane damage 18, 19. Uncontrolled lipid peroxidation is a significant feature of ferroptosis, resulting from the interaction between the ferroptosis-inducing and defense systems 19. Ferroptosis is activated when the promoters of ferroptosis significantly exceed the antioxidant capacity of the defense system 19. Some oncogenes and oncogenic signaling can activate the antioxidant or ferroptosis defense system, favoring tumorigenesis, progression, metastasis, and resistance 20, 21. Therefore, this study aimed to analyze the expression of EPS15-AS1 and EPS15 in LIHC and to investigate whether EPS15-AS1 has the ability to regulate EPS15 and the sensitivity of LIHC to ferroptosis.

## Materials and Methods

### Cell Culture

A total of three cell lines, Huh7, HepG2, and HL7702, were used in the current study. HL7702 is a normal human hepatocyte cell line, and Huh7 and HepG2 are human LIHC cell lines. All cell lines were purchased from the Shanghai Institute of Biochemistry and Cell Biology (SIBCB) and cultured in Dulbecco's modified Eagle's medium (DMEM) (HyClone, USA) containing 10% fetal bovine serum (FBS) (Gibco, USA). All cells were cultured at 37 °C and 5% CO2 in a humid incubator (Thermo Fisher, USA). When the cultured cells were fused to 80-90%, cells were digested with 0.25% trypsin (NCM Biotech, China) and passaged in 1 to 3 passages.

### Western Blot Analysis

After incubation under different intervention conditions, all cells were collected and lysed using RIPA lysis buffer (NCM Biotech, China). After 3 minutes of lysis, the lysates were centrifuged at 12,000 rpm for 10 minutes, and the supernatant was collected for western blot analysis. The protein concentrations were quantified using the BCA kit (NCM Biotech, China) to keep the total amount of protein consistent across the different experimental groups. Finally, 20 μg of protein per group was used for western blot analysis. 10% sodium dodecyl sulfate-polyacrylamide gel electrophoresis (10% SDS-PAGE) (Vazyme, China) was applied to separate the protein, and then the protein was transferred to nitrocellulose membranes (Millipore, USA) at 300 mA for 1 hour. The nitrocellulose membranes containing protein were blocked with 5% nonfat powdered milk (Beyotime, China). Then, the membranes were incubated with the corresponding primary antibody at 4 °C for 12 h. The primary antibodies against EPS15 (dilution ratio, 1:1000), β-Actin (dilution ratio, 1:20000) and AKR1B1 (dilution ratio 1:1000) were purchased from ABclonal (#A9814, #AC038, #A18031). Next, the nitrocellulose membranes were washed with TBS-Tween and incubated with secondary antibody (HRP-conjugated goat anti-rabbit IgG, ABclonal, #AS014, 1:10,000). Finally, the chemiluminescent HRP substrate (NCM Biotech, China) was applied for imaging, and the image was detected by a chemiluminescence detection system (Bio-Rad, USA).

### Real-Time Quantitative PCR Analysis (RT‒qPCR)

In the current study, total RNA was collected with a total RNA isolation kit (Vazyme, # RC101-01), and 500 ng of RNA was reverse transcribed into cDNA using RT SuperMix (Vazyme, #R233-01). SYBR qPCR Master Mix (Vazyme, #Q321-02) was applied to perform RT‒qPCR, and GAPDH was chosen as an internal control. Then, the 2^-(∆∆Ct) method was used to calculate the relative expression levels of EPS15-AS1 and EPS15. The reaction conditions were set as follows: 95.0 °C for 3 minutes and then 40 cycles of 95.0 °C for 5 seconds, 60.0 °C for 30 seconds and 72.0 °C for 30 seconds. All primer sequences were as follows:

GAPDH

F: 5'-CATCACTGCCACCCAGAAGACTG-3'

R: 5'-ATGCCAGTGAGCTTCCCGTTCAG-3';

EPS15

F: 5'-ACCTTCACTTAGGCCCCTGT-3'

R: 5'-CCCTTACCCTCACTCAACCA-3';

EPS15-AS1

F: 5'-ACCCCAAAGCCTCTTGATTT-3'

R: 5'-CGTCTCCTCAGACGGTTCTC-3'.

### Invasion Assay

The trans-well chamber used in the current study was purchased from NEST Biotech (China, #725201). HepG2 cells were digested and resuspended at a concentration of 200,000/ml, and then, 100 μL of the cell suspension was seeded in each upper chamber of the trans-well. In addition, 500 μL of DMEM containing 10% FBS was added to the lower chamber of the trans-well. Finally, the cells were cultured for 24 hours, and the trans-well chamber with cells was collected and stained with 0.1% crystal violet solution.

### Wound Healing Assay

HepG2 cells (1 × 10^5^ per well) were seeded in 6-well plates for the migration assay. Until the cells were fused to 90-95%, the monolayer of cells was scratched with a 200 μL plastic tip. Then, the cells were rinsed three times with PBS and cultured with DMEM containing 5% FBS for 12 hours. Images were taken at 0 and 12 hours for analysis of migration distance: migration distance = (initial wound width - wound width at each time point)/2 (μm).

### Flow Cytometry

Mitochondrial membrane potential staining was performed using the JC-1 staining kit, which was purchased from Beyotime Biotech, China (#C2006). Lipid peroxidation was detected using a lipid peroxidation probe-BDP 581/591 C11 kit (Dojindo, Japan, #L267). In addition, intracellular Fe^2+^ ions were detected with an iron ion detection probe-FerroOrange kit (Dojindo, #F374), and an Annexin V-FITC/PI Kit (Dojindo, #AD10) was used to detect the percentage of cells with damaged cell membranes. All staining was performed according to the corresponding manufacturer's instructions.

### Transfection and Construction of Overexpression Cell Lines

The expression vector used in the current study was pcDNA3.1, and Lipofectamine 2000 (Thermo Fisher, USA) was used to transfect pcDNA3.1. In addition, the three overexpression plasmids, including overexpression EPS15 (OE_EPS15), overexpression EPS15-AS1 (OE_EPS15-AS1), and overexpression AKR1B1 (OE_AKR1B1), were all purchased from Sangon Biotech (Shanghai, China). The overexpression plasmids and Lipofectamine 2000 were mixed separately with 50 µl of DMEM and left to stand for 5 minutes. Then, the plasmid and Lipofectamine 2000 were mixed and incubated for 20 minutes at room temperature, and the transfection complex was immediately added to the HepG2 culture plate. Then, the HepG2 cells and plasmids were cultured together for 24 hours, switched to normal DMEM containing 10% FBS, and cultured for another 24 hours. After obtaining overexpression cell lines, gene expression levels were examined using RT‒qPCR and western blot analysis.

### Online Databases and Bioinformatics Analysis

The GEPIA2 online analysis tool (http://gepia2.cancer-pku.cn/#index) is a tool for analyzing The Cancer Genome Atlas (TCGA) database and was used to perform survival analysis and to compare the expression of EPS15 and AKR1B1 in LIHC tissues and adjacent normal tissues.

To find the correlation between EPS15 and ferroptosis-associated proteins, an interaction network between EPS15 and ferroptosis-associated proteins was constructed by using STRING (https://cn.string-db.org/), which is a protein‒protein interaction network functional enrichment analysis website. In addition, ferroptosis-associated protein was obtained from FerrDb (http://www.zhounan.org/ferrdb/current/), a database that summarizes the latest ferroptosis-associated markers and genes. Cytoscape 3.10.0 was used to show the interaction network diagram.

### Statistical Analysis

GraphPad Prism (version 9.0) was applied to conduct statistical analysis. The mean ± standard deviation was calculated to describe continuous variables. A t test was used to compare the two groups, and one-way ANOVA followed by Dunnett's multiple comparisons test was used for statistical analysis among multiple groups. P < 0.05 was considered to indicate a significant difference.

## Results

### EPS15-AS1 expression was decreased in LIHC cells

**Figure 1:**
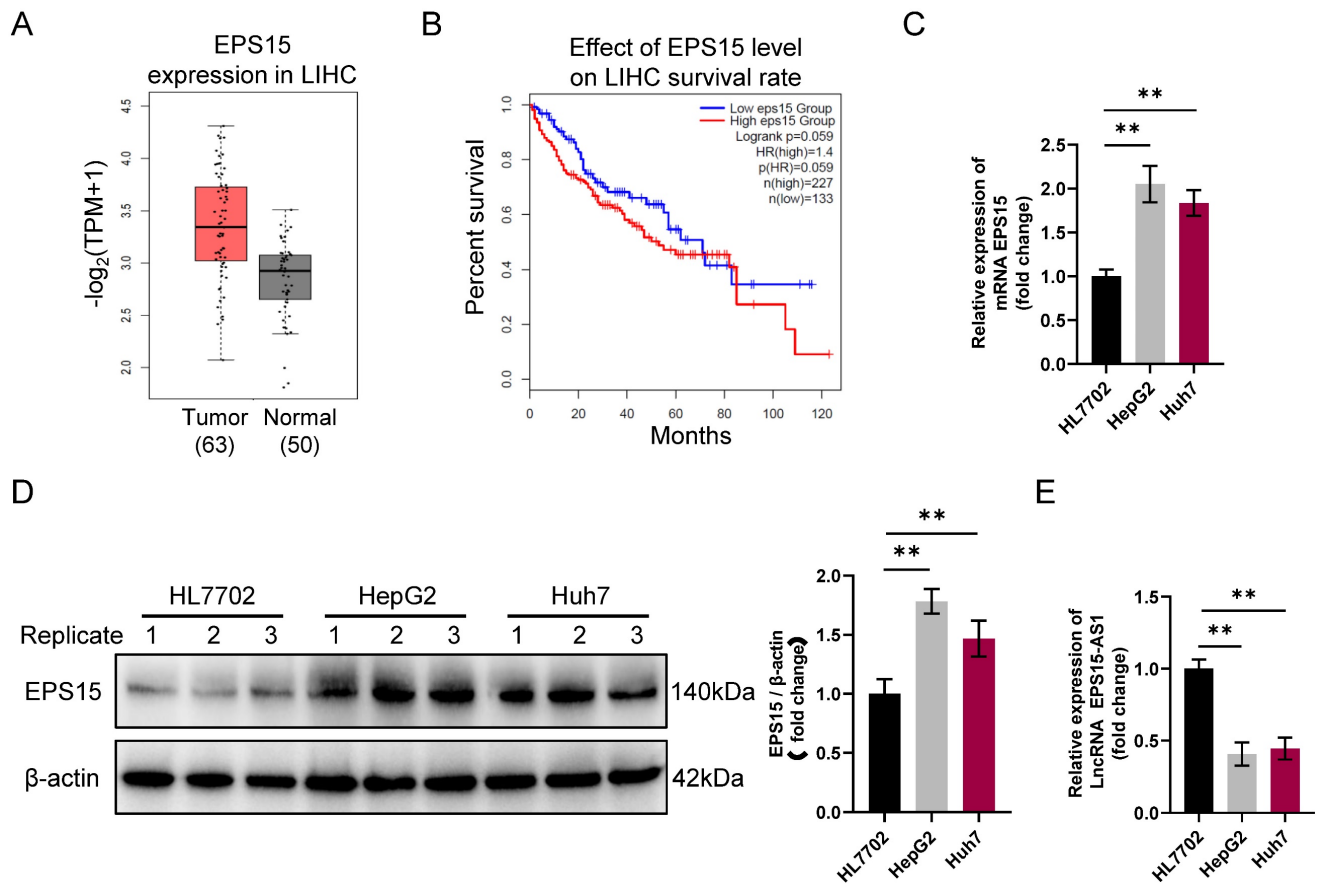
Differential expression of EPS15 and EPS15-AS1 in liver cancer and normal liver tissue. (A) Comparison of EPS15 expression in hepatocellular carcinoma and normal tissues from the TCGA cancer genome database (P=0.055). (B) Kaplan‒Meier survival analysis of patients with high and low expression of EPS15 (log rank p=0.059). (C) RT‒qPCR analysis of EPS15 expression in HL7702, HepG2 and Huh7 cell lines (GAPDH was set as internal control). (D) Western blot analysis of EPS15 expression in HL7702, HepG2 and Huh7 cell lines (β-actin was set as internal control). (E) RT‒qPCR analysis of LncRNA EPS15-AS1 expression. (*p<0.05 and **p<0.01, n=3 each group).

The EGFR signaling pathway is one of mammalian cell physiology's most important signaling pathways 10. It promotes tumorigenesis mainly by affecting tumor cell proliferation, angiogenesis, tumor invasion, and metastasis. Aberrant activation of EGFR signaling pathways is one of the mechanisms of tumor development. It has been reported that the EPS15 gene encodes a protein part of the EGFR signaling pathway 12. In this study, according to the data of LIHC tissues in the TCGA database, we observed that the expression of EPS15 in LIHC tissue was higher than that in normal liver tissue (p = 0.055) (**Figure 1A**).

Additionally, the patients with high expression of EPS15 had a lower survival rate than those with low EPS15 expression (log rank p = 0.059) (**Figure 1B**). Comparison between the normal hepatocyte cell line HL7702 and the LIHC cell lines HepG2 and Huh7 further revealed that the gene transcription and protein expression levels of EPS15 were higher in LIHC cells than in normal hepatocyte cells (**Figure 1C** and** 1D**). Interestingly, we also found that the transcription level of the lncRNA EPS15-AS1 was significantly decreased in HepG2 and Huh7 cells compared with that in HL7702 cells (**Figure 1E**). Therefore, these results suggest that the expression level of EPS15 is closely related to the development of LIHC and that EPS15-AS1 may be involved in the regulation of EPS15 expression during the development of LIHC.

### EPS15-AS1 inhibited LIHC cell activity by decreasing EPS15 expression

**Figure 2:**
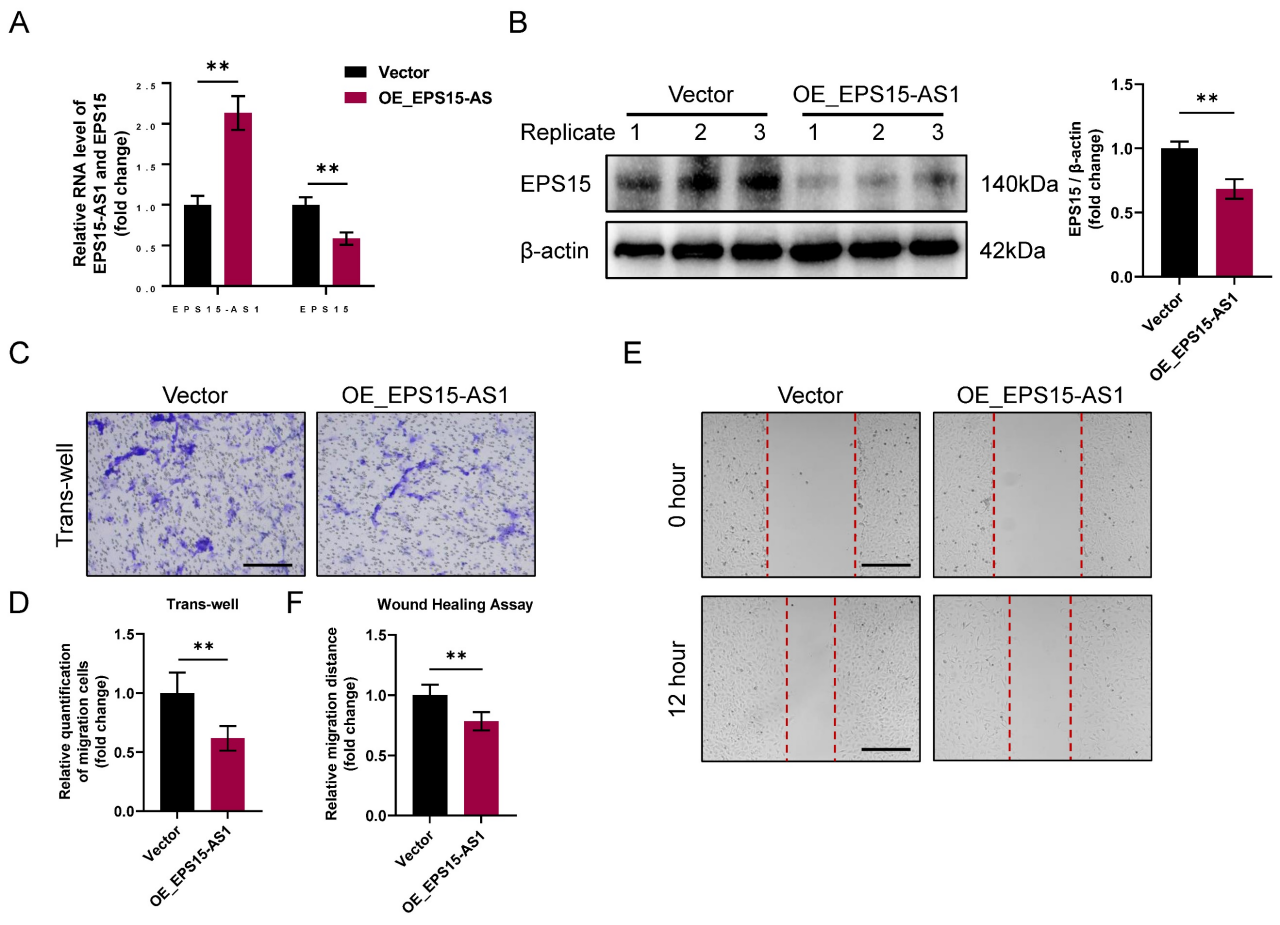
Overexpression of EPS15-AS1 reduced EPS15 expression in HepG2 and inhibited cell migration. (A) RT‒qPCR analysis of EPS15-AS1 and EPS15 in HepG2 with EPS15-AS1 overexpression. (B) Western blot analysis of EPS15. (C) Invasion assay. (Scale bar: 100 μm) (D) The number of cells passing through the upper chamber of the trans-well. (E) Wound healing assay. (Scale bar: 200 μm) (F) The migration distance in the wound healing assay. (*p<0.05 and **p<0.01, n=3 each group).

Antisense lncRNAs are transcribed from the antisense strand of a protein-coding gene and overlap with the mRNA of the gene, and this structure of antisense lncRNAs provides the basis for the regulation of gene expression 16. Thus, we hypothesize that EPS15-AS1 can modulate EPS15 expression in LIHC cells, which in turn affects the invasiveness of LIHC cells. To verify the effects of EPS15-AS1 in HepG2, overexpression of EPS15-AS1 was performed using the pcDNA3.1 plasmid. RT‒qPCR and western blotting analysis showed that EPS15 transcripts were significantly reduced in the EPS15-AS1 overexpression group (OE_EPS15-AS1), and the level of EPS15 proteins was also decreased (**Figure 2A** and** 2B**). In addition, invasion assays showed that the number of cells passing through the trans-well chambers was reduced in the OE_EPS15-AS1 group (**Figure 2C** and** 2D**), and wound healing assays also showed a significant decrease in the migratory ability of the OE_EPS15-AS1 group compared with the control group (vector group) (**Figure 2E** and** 2F**). These results suggest that EPS15-AS1 can inhibit LIHC cell activity by affecting the expression of EPS15.

**Figure 3:**
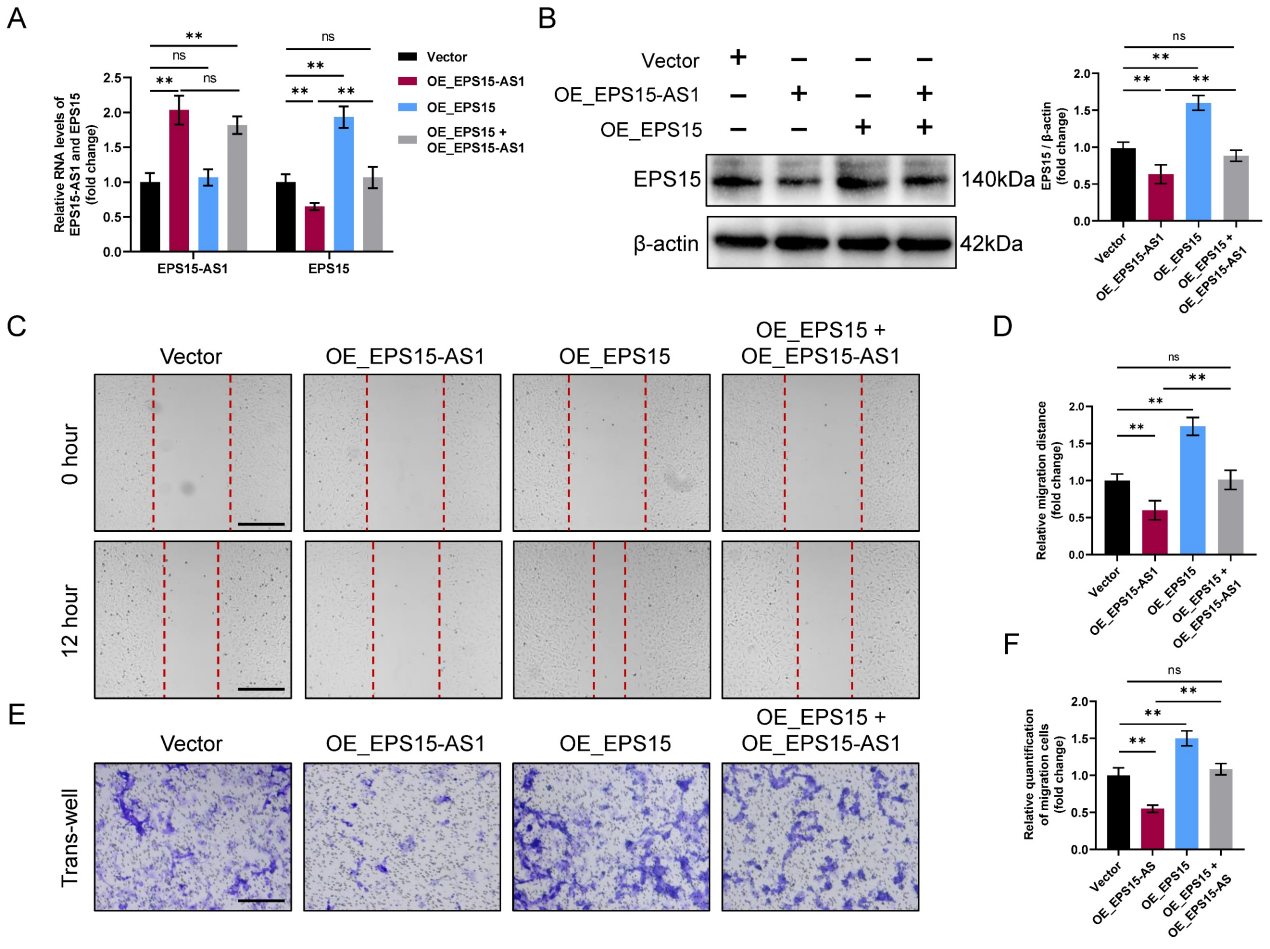
EPS15-AS1 alleviated the promoting effect of EPS15 on cell invasiveness. (A) RT‒qPCR analysis of EPS15-AS1 and EPS15 in HepG2 after overexpression of EPS15-AS1 and/or EPS15. (B) Western blot analysis of EPS15. (C) Wound healing assay. (Scale bar: 200 μm) (D) The migration distance in the wound healing assay. (E) Invasion assay. (Scale bar: 100 μm) (F) The number of cells passing through the upper chamber of the trans-well. (*p<0.05 and **p<0.01, ns: no significance, n=3 each group).

Next, to verify whether EPS15-AS1 inhibits LIHC cell activity mainly by affecting EPS15 expression, we overexpressed EPS15 and EPS15-AS1 together. In the following experiment, the intervention was divided into four groups: (1) Vector (control group), (2) OE_EPS15-AS1 (EPS15-AS1 overexpression group), (3) OE_EPS15 (EPS15 overexpression group) and (4) OE_EPS15 + OE_EPS15-AS1 (EPS15 and EPS15-AS1 overexpression group). RT‒qPCR results showed that EPS15 expression increased in the OE_EPS15 group and decreased in the OE_EPS15-AS1 group (**Figure 3A**). In the OE_EPS15 + OE_EPS15-AS1 group, we found that the expression of EPS15 was not significantly different from that in the vector group (**Figure 3A**). Moreover, western blot analysis further validated the RT‒qPCR results that EPS15 was not significantly different between the vector group and the OE_EPS15 + OE_EPS15-AS1 group (**Figure 3B**). Finally, both the invasion and wound healing assays confirmed that elevated EPS15 promoted the invasiveness of hepatocellular carcinoma, but overexpression of EPS15-AS1 inhibited HepG2 activity by suppressing EPS15 expression (**Figure 3C-F**). Therefore, all these results suggest that EPS15 has the ability to promote LIHC cell invasiveness, whereas overexpression of EPS15-AS1 can inhibit LIHC cell activity and invasiveness by downregulating EPS15 expression.

### EPS15-AS1 increases the susceptibility of LIHC cells to ferroptosis

**Figure 4:**
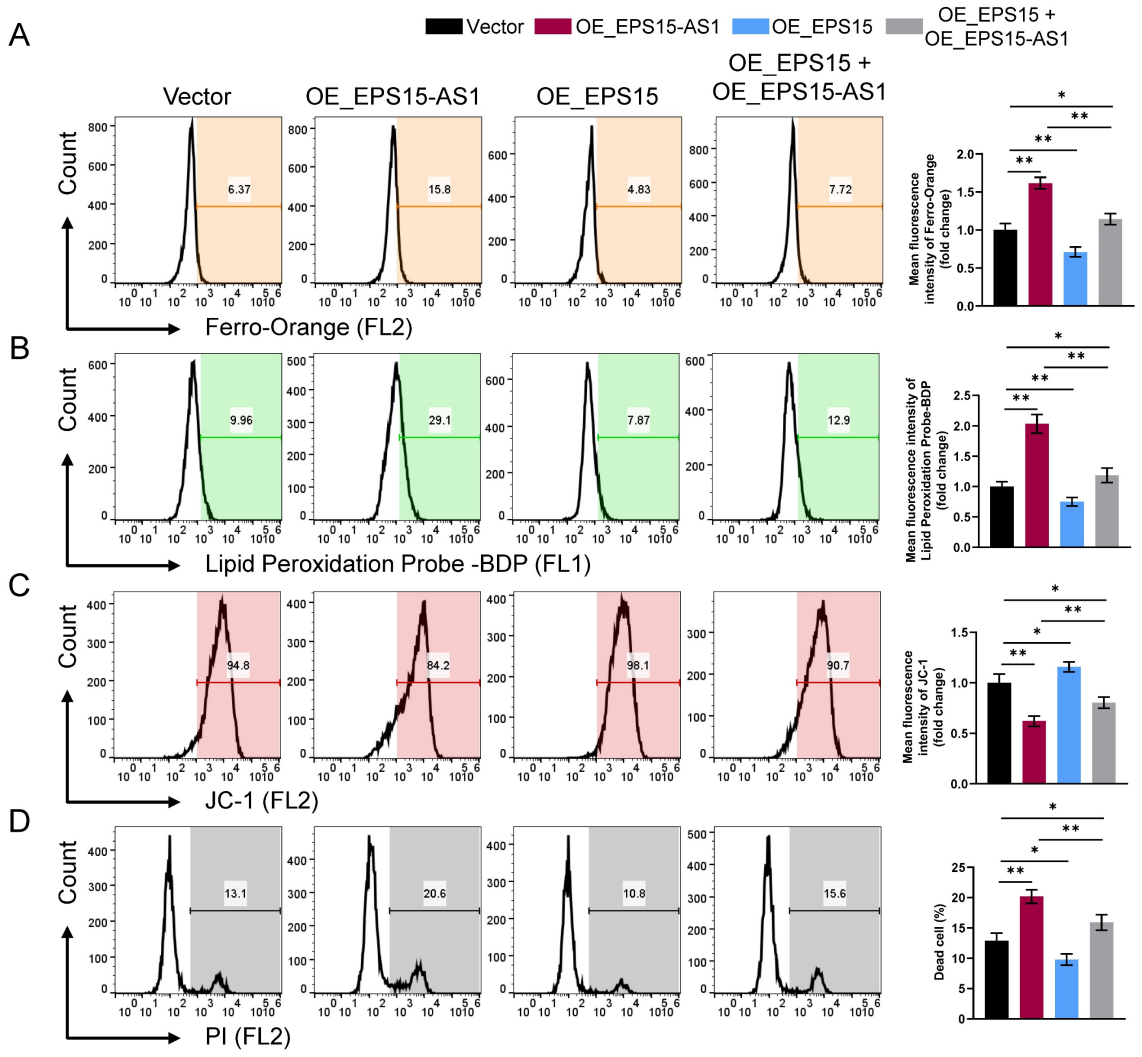
EPS15-AS1 induced ferroptosis in HepG2 cells by inhibiting EPS15 expression. (A) Fe^2+^ was detected with a Ferro-Orange fluorescent probe. (B) Fluorescent probe-BDP was used to analyze lipid peroxidation. (C) Mitochondrial membrane potential was analyzed with the JC-1 fluorescent probe. (D) Dead cells were detected using the nucleic acid dye propidium iodide (PI). (*p<0.05 and **p<0.01, n=3 each group).

During the previous experiments, we observed that the cellular status became significantly worse with overexpression of EPS15-AS1 or inhibition of EPS15 expression. Moreover, we also found significant changes in intracellular Fe^2+^ ion levels (**Figure 4A**), leading us to suspect that EPS15 may influence the relationship between LIHC and ferroptosis. As shown in **Figure 4A**, intracellular Fe^2+^ increased in the OE_EPS15-AS1 group and decreased in the OE_EPS15 group compared with the Vector group. Ferroptosis is an iron-dependent programmed cell death characterized by mitochondrial dysfunction and uncontrolled lipid peroxidation. JC-1 is a mitochondrial membrane potential staining reagent, and BDP is a lipid peroxidation probe. As shown in **Figures 4B** and **4C**, overexpression of EPS15-AS1 significantly promoted lipid peroxidation and mitochondrial dysfunction in HepG2 cells, whereas overexpression of EPS15 attenuated the effects of EPS15-AS1. Finally, to observe whether the changes in mitochondria and lipids would eventually lead to cell death, propidium iodide (PI) staining was performed. PI is an agent that can bind to DNA and usually cannot pass through normal living cell membranes but can pass through damaged cell membranes or dead cells. As shown in **Figure 4D**, overexpression of EPS15-AS1 led to ferroptosis in LIHC cells, whereas expression of EPS15 alleviated the ferroptosis induced by overexpression of EPS15-AS1. Moreover, when OE_EPS15-AS1 cells were treated with ferroptosis inhibitors Ferrostain-1 and Deferasirox, the percentage of dead cells was decreased in the Ferrostain-1 and Deferasirox groups (**Figure S1**). These results indicated that EPS15-AS1 increases the susceptibility of LIHC cells to ferroptosis by inhibiting the transcription of EPS15.

### EPS15 enhances LIHC cell activity by promoting the expression of AKR1B1

**Figure 5:**
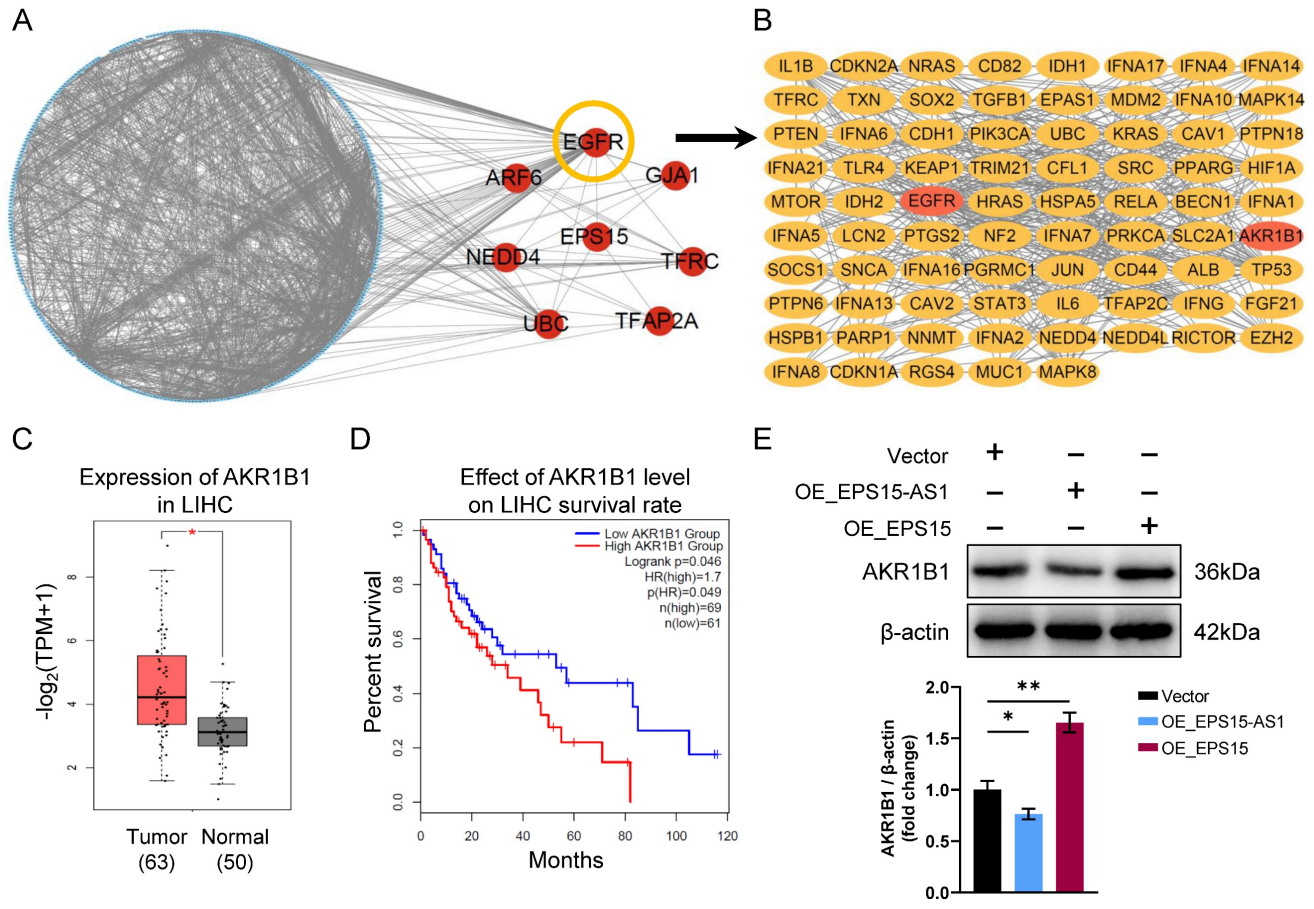
AKR1B1 is involved in the promotion effects of EPS15 on LIHC. (A) Interaction network between EPS15 and ferroptosis-associated proteins. (B) The subnetwork of EGFR and ferroptosis-associated proteins was extracted from the EPS15 network. (C) AKR1B1 expression in hepatocellular carcinoma and normal tissues from the TCGA cancer genome database. (D) Kaplan‒Meier survival analysis of patients with high and low expression of AKR1B1. (E) Western blot analysis of AKR1B1. (*p<0.05 and **p<0.01, n=3 each group).

To investigate the mechanism between EPS15 and ferroptosis, an interaction network between EPS15 and ferroptosis-associated proteins was constructed (**Figure 5A**), and according to the interaction network, EGFR, ARF6, GJA1, NEDD4, TFRC, UBC, and TFAP2A were significantly correlated with EPS15 (marked by red circles in **Figure 5A**). Interestingly, EGFR is also a ferroptosis-related protein and is associated with a large number of other ferroptosis-associated proteins in the network, as shown in **Figure 5A**, where interacting straight lines cluster around EGFR. We then constructed a subnetwork consisting of EGFR-associated proteins from the network of Figure 5A (**Figure 5B**).

The aldo-keto reductase family 1 member B1 (AKR1B1) gene encodes a member of the aldo/keto reductase superfamily, and this gene catalyzes the reduction of a number of aldehydes 22. Recently, AKR1B1 was reported to promote drug resistance to EGFR TKIs in lung cancer cell lines 23. The current study also found that AKR1B1 correlates with EPS15 and EGFR (**Figure 5B**). In addition, we observed that in the TCGA database, the expression of AKR1B1 in LIHC was higher than that in normal tissue (p < 0.05) (**Figure 5C**). The patients with high AKR1B1 expression had a lower survival rate than the patients with low AKR1B1 expression (log-rank p < 0.05) (**Figure 5D**). Then, we further found that overexpression of EPS15 in LIHC increased the expression of AKR1B1 by using western blotting analysis, whereas the expression of AKR1B1 was reduced in the OE_EPS15-AS1 group (**Figure 5E**). Therefore, we conclude that EPS15 can promote AKR1B1 expression in LIHC.

**Figure 6:**
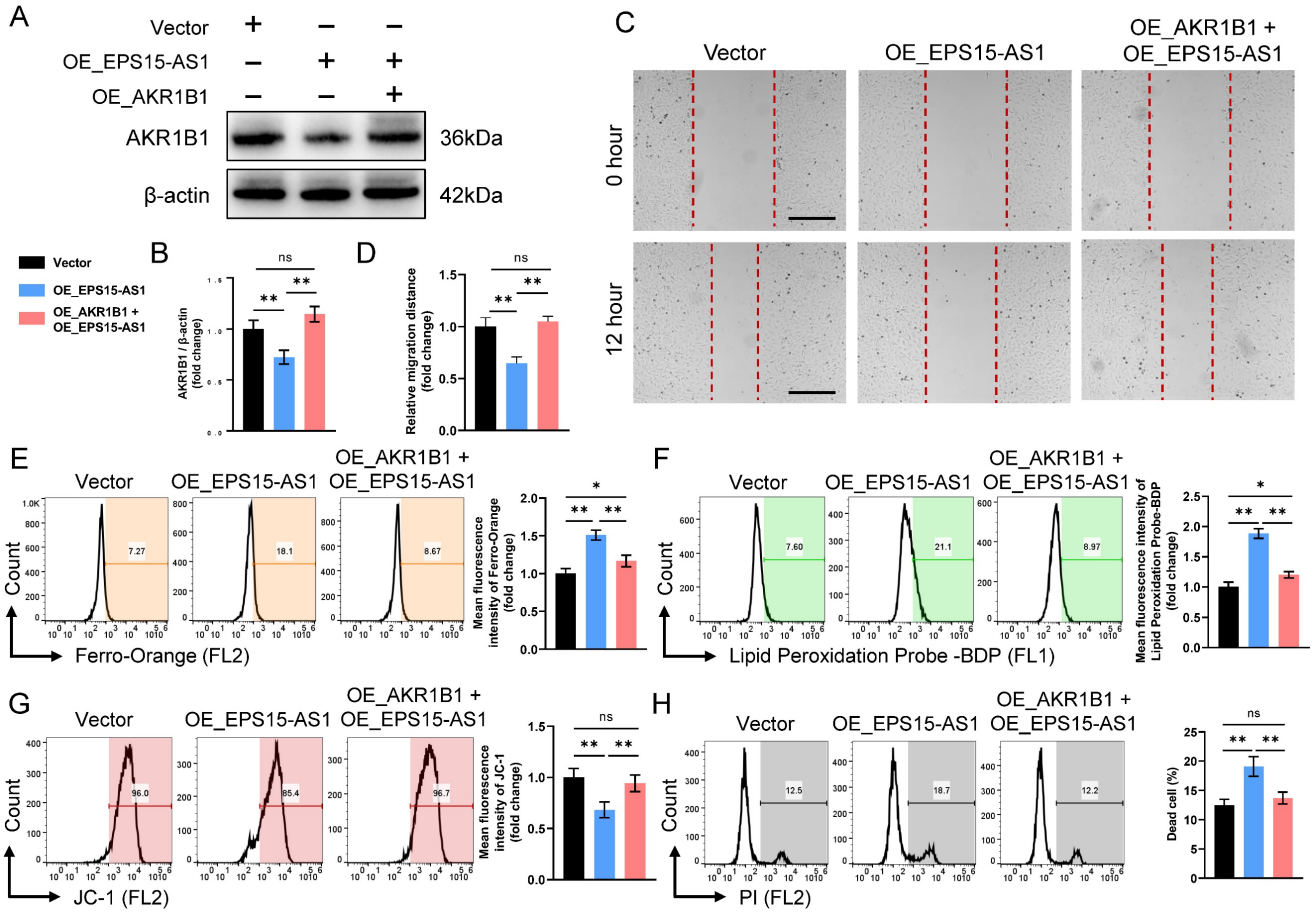
EPS15 enhanced cell invasiveness by promoting AKR1B1 expression. (A) and (B) Western blot analysis of AKR1B1. (C) Wound healing assay. (Scale bar: 200 μm) (D) The migration distance in the wound healing assay. (E) Fe^2+^ was detected with a Ferro-Orange fluorescent probe. (F) Lipid peroxidation was detected with the fluorescent probe-BDP. (G) Mitochondrial membrane potential was analyzed with the JC-1 fluorescent probe. (H) Dead cells were detected with PI. (*p<0.05 and **p<0.01, ns: no significance, n=3 each group).

To further clarify whether EPS15 promotes LIHC development through AKR1B1, we constructed OE_EPS15-AS1 HepG2 cell lines and OE_EPS15-AS1 + OE_AKR1B1 HepG2 cell lines in LIHC (**Figure 6A**). Although EPS15-AS1 inhibited cell migration in wound healing assays, overexpression of AKR1B1 reversed the inhibitory effect of EPS15-AS1 (**Figure 6B**). In the Fe^2+^ detection assay, overexpression of AKR1B1 significantly reduced the elevated Fe^2+^ caused by overexpression of EPS15-AS1 (**Figure 6C**). Detection of lipid peroxidation and mitochondrial membrane potential also confirmed that EPS15-AS1 enhanced lipid peroxidation and disrupted mitochondrial membrane potential, and overexpression of AKR1B1 significantly inhibited this damage (**Figure 6D** and** 6E**). PI staining further demonstrated that AKR1B1 reduced the ratio of dead cells in the OE_EPS15-AS1 + OE_AKR1B1 group compared with the OE_EPS15-AS1 group (**Figure 6F**). In addition, Zhang et al. reported that AKR1B1 promotes glutathione (GSH) de novo synthesis to protects against oxidative damage, and glutathione peroxidase 4 (GPX4) is able to utilize GSH to reduce peroxidized lipids to non-toxic lipids, thereby protecting cells from ferroptosis 22. Therefore, the intracellular GSH was also detected. The results showed that intracellular GSH decreased after EPS-AS1 overexpression, increased in OE_AKR1B1 group, and the inhibitory effect of EPS-AS1 on GSH was attenuated in OE_EPS15-AS1 + OE_AKR1B1 group (**Figure S2**). These results suggested that AKR1B1 can promote LIHC progression and that EPS15-AS1 increases the susceptibility of LIHC cells to ferroptosis by inhibiting the transcription of EPS15 and AKR1B1.

## Discussion

LIHC is one of the most common malignant tumors, but metastasis and postoperative recurrence seriously affect the long-term prognosis 3. In addition, resistance to chemotherapeutic agents is an important reason for the low efficacy of radiotherapy and chemotherapy in hepatocellular carcinoma patients 24. Therefore, an increasing number of researchers believe that combination gene therapy may be a potential direction for the treatment of LIHC 25.

Approximately 90% of genes in eukaryotic genomes are transcribed, with only 1-2% of transcribed genes coding for proteins, while most other genes are transcribed as noncoding RNAs 26, 27. Noncoding RNAs play an important role at the transcriptional and posttranscriptional levels of encoded genes 27. In the current study, we found that EPS15 was closely associated with the progression of LIHC by analyzing the TCGA database. With further analysis, we found that the expression level of EPS15-AS1 was reduced in LIHC cells. Liu et al. also found that EPS15-AS1 was expressed at low levels in liver cancer cells, and overexpression of EPS15-AS1 reduced EPS15 expression and promoted apoptosis of liver cancer cells 17. The current study showed that EPS15 was increased in LIHC cell lines, including HepG2 and Huh7, compared with the normal hepatocyte cell line HL7702. We also demonstrated that overexpression of EPS15-AS1 inhibited EPS15 expression and weakened the invasiveness of hepatocellular carcinoma cell lines.

However, we found that overexpression of EPS15-AS1 induced ferroptosis but not apoptosis in LIHC cells. This difference in conclusions may be due to the different experimental methods used to detect cell death between the two studies. Annexin V-FITC/PI was initially invented to detect the process of apoptosis 28. The mechanism of this assay is as follows: in living cells, phosphatidylserine (PS) is located on the inner side of the cell membrane, but in early apoptotic cells, the PS flips from the inner side of the cell membrane to the surface of the cell membrane. Annexin-V, a Ca^2+^-dependent PS-binding protein, can bind to the cell membrane during the early stage of apoptosis by binding to the PS exposed outside of cells. In the late stage of apoptosis, the cell membrane is severely damaged, and Annexin-V can freely pass through the cell membrane 28. In addition, propidium iodide (PI) was used to distinguish surviving cells from necrotic or late-stage apoptotic cells. PI is a nucleic acid dye that does not pass through the intact cell membranes of normal or early apoptotic cells but can pass through the cell membranes of late apoptotic and necrotic cells and stain the cell nucleus 28. Therefore, PI is excluded from living cells (Annexin V-/PI-) and early apoptotic cells (Annexin V+/PI-), while late apoptotic and necrotic cells are stained double-positive (Annexin V+/PI+). Interestingly, during ferroptosis, cell membranes are subjected to uncontrolled lipid peroxidation, ultimately causing cell membrane disruption. Finally, cells undergoing ferroptosis were stained double-positive (Annexin V+/PI+). Thus, Annexin V-FITC/PI cannot distinguish apoptosis and ferroptosis, and other experiments are needed for additional validation.

In the current study, we further examined intracellular Fe^2+^, lipid peroxidation, and mitochondrial membrane potential to determine what kind of cell death is involved. After overexpression of EPS15-AS1, intracellular Fe^2+^ and lipid peroxidation were enhanced, and mitochondrial membrane potential was disrupted. Moreover, co-overexpression of EPS15-AS1 and EPS15 attenuated the damaging effects of EPS15-AS1. With bioinformatic analysis, we further found that AKR1B1, which can influence ferroptosis, was associated with EPS15. AKR1B1 was overexpressed in LIHC cells, and overexpression of EPS15-AS1 inhibited AKR1B1 expression. Moreover, overexpression of EPS15-AS1 and AKR1B1 in HepG2 cells showed similar invasiveness to normal HepG2 cells and had normal levels of Fe^2+^, lipid peroxidation, and mitochondrial membrane potential. This confirmed that AKR1B1 can promote LIHC cell activity against ferroptosis. In addition, Zhang et al. also reported that AKR1B1 has the ability to promote resistance to EGFR-targeted therapy in lung cancer by enhancing glutathione de novo synthesis 23.

However, the current study had some limitations as well. The mechanism by which EPS15 promotes AKR1B1 is still unclear. Furthermore, whether AKR1B1 also promotes LIHC cell activity by facilitating the glutathione de novo synthesis is unknown. Therefore, future studies should further clarify the exact mechanisms of EPS15 and AKR1B1 promoting hepatocellular carcinoma.

## Conclusion

In conclusion, the current study showed that EPS15-AS1 expression had an inhibitory effect on hepatocellular carcinoma. Further investigation demonstrated that EPS15-AS1 reduced EPS15 expression and thus downregulated AKR1B1 expression, which finally inhibited the invasiveness of LIHC cells and induced ferroptosis in LIHC. In general, EPS15-AS1 may be a candidate target for hepatocellular carcinoma and may be a therapeutic strategy to overcome drug resistance.

## Supplementary Material

Supplementary figures.

## Abbreviations

EPS15: epidermal growth factor receptor substrate 15
LncRNA: long noncoding RNA
LIHC: liver hepatocellular carcinoma
TCGA: the cancer genome atlas
EPS15-AS1: lncRNA EPS15-antisense1
AKR1B1: Aldo-keto reductase family 1 member B 1
RTKs: receptor tyrosine kinases
EGFR: epidermal growth factor receptor
DMEM: Dulbecco's modified Eagle's medium
FBS: fetal bovine serum
RT‒qPCR: Real-Time Quantitative PCR Analysis
