# Supplementary material for: EPS15-AS1 Inhibits AKR1B1 Expression to Enhance Ferroptosis in Hepatocellular Carcinoma Cells
Source: J Cancer. 2024 Jan 1;15(4):1030–40. doi: 10.7150/jca.89993 (PMC10788721; doi:10.7150/jca.89993)
Supplement: Supplementary file 1 — Supplementary figures. [file jcav15p1030s1.pdf]

Supplementary Materials

EPS15-AS1 Inhibits AKR1B1 Expression to Enhance Ferroptosis in Hepatocellular Carcinoma Cells

Quan Man[a], Guoyou Zhang[a], Xiaojun Chen[b], Sa Ren Na[c], Siguleng Bai[a], Haoqiang Zhi[b], Ling Sun[b], Huifang Pang[b, *]

*** Correspondence:** Huifang Pang, Email: phf1986inner@163.com

Supplementary Figures

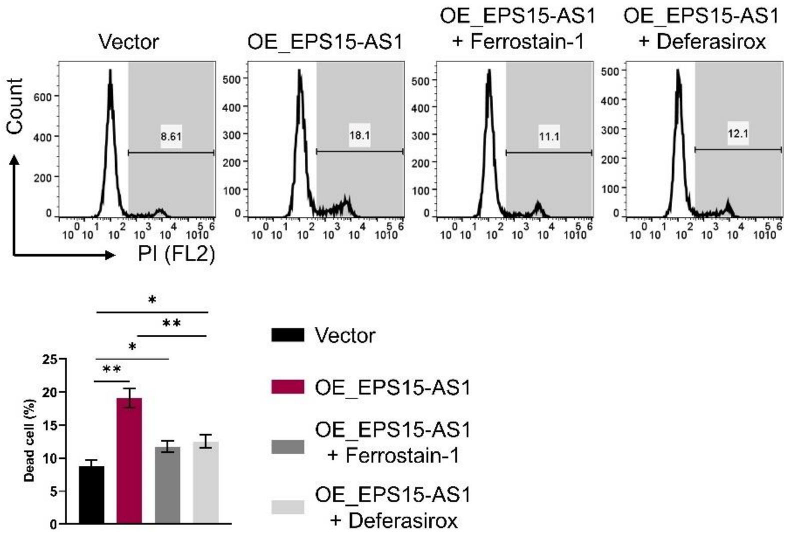

Figure S1 Percentage of dead cells were detected using the nucleic acid dye propidium iodide (PI). (*p<0.05 and **p<0.01, n=3 each group).

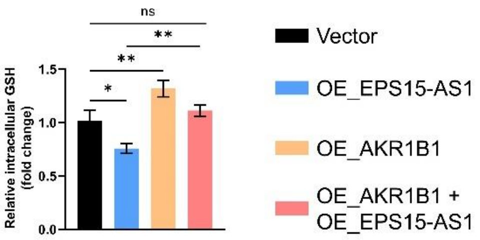

Figure S2 Intracellular GSH. (*p<0.05 and **p<0.01, n=3 each group).
